# Determinants of malaria diagnostic uptake in the retail sector: qualitative analysis from focus groups in Uganda

**DOI:** 10.1186/s12936-015-0590-x

**Published:** 2015-02-21

**Authors:** Jessica Cohen, Alex Cox, William Dickens, Kathleen Maloney, Felix Lam, Günther Fink

**Affiliations:** Department of Global Health and Population, Harvard School of Public Health, Boston, MA USA; Department of Economics, Northeastern University, Boston, MA USA; Malaria Control Team, Clinton Health Access Initiative, Boston, MA USA; Essential Medicines Team, Clinton Health Access Initiative, Boston, MA USA

**Keywords:** Malaria, Rapid diagnostic test, RDT, Diagnostics, Drug shop, Private sector, Acceptability, Uptake, Uganda

## Abstract

**Background:**

In Uganda, as in most other malaria-endemic countries, presumptive treatment for malaria based on symptoms without a diagnostic blood test is still very common. While diagnostic testing in public sector facilities is increasing, many people in Uganda who suspect malaria visit private sector outlets to purchase medications. Increasing the availability and uptake of rapid diagnostic tests (RDTs) for malaria in private outlets could help increase diagnostic testing for malaria but raises questions about the patient demand for and valuation of testing that are less critical for public sector introduction.

**Methods:**

In preparation for a behaviour change campaign to encourage and sustain the demand for RDTs in drug shops, eight focus group discussions with a total of 84 community members were conducted in six districts across Uganda’s Eastern Region in November-December 2011. Focus groups explored incentives and barriers to seeking diagnosis for malaria, how people react to test results and why, and what can be done to increase the willingness to pay for RDTs.

**Results:**

Overall, participants were very familiar with malaria diagnostic testing and understood its importance, yet when faced with limited financial resources, patients preferred to spend their money on medication and sought testing only when presumptive treatment proved ineffective. While side effects did seem to be a concern, participants did not mention other potential costs of taking unnecessary or ineffective medications, such as money wasted on excess drugs or delays in resolution of symptoms. Very few individuals were familiar with RDTs.

**Conclusion:**

In order to boost demand, these results suggest that private sector RDTs will have to be made convenient and affordable and that targeted behaviour change campaigns should strive to increase the perceived value of diagnosis.

## Background

The continued reliance on presumptive treatment for malaria means that many patients receive an incorrect diagnosis of malaria and inappropriate treatment for the actual cause of their illness [1-3]. In addition to poor patient management, presumptive treatment and the ensuing over-treatment of malaria with artemisinin-based anti-malarials represents a waste of private and public resources and could increase the risk of the emergence of resistant parasite strains [4,5]. In response, in 2010, the World Health Organization (WHO) changed its fever case management guidelines to recommend that artemisinin-based combination therapy (ACT) be used only in patients that have a blood test diagnosis of malaria when possible. Following this guideline change, many countries aligned their national strategy with the WHO recommendation and began to scale-up access to diagnostic technology in their public sector [6].

Great progress has been made in the public sector; confirmation of malaria cases in the public sector has increased from less than 10 to over 60% between 2000 and 2013 in the WHO African Region [7]. However, in the private sector, uptake of malaria diagnostics has remained at much lower levels [7,8]. A study from six African countries found that only 2 to 16% of children with febrile illnesses received a malaria blood test in the private sector prior to treatment [9]. Purchasing over-the-counter medication from a pharmacy or drug shop without first seeking professional medical advice is a very common response to fever episodes in sub-Saharan Africa [9-11]. Treatment-seeking in the retail sector is common because health facilities are often distant, with limited staff, long wait times, limited hours, and frequent stock-outs of essential medicines. Even when fees at public health facilities are low, visits to private or informal drug shops are common due to their convenience and accessibility [12].

The heavy reliance on drug shops and other private outlets has led the malaria community to consider ways of leveraging this sector to improve fever case management [13,14]. The Affordable Medicines Facility-malaria (AMFm) initiative, piloted in seven countries in 2010, is one example. The AMFm was an innovative financing mechanism designed to increase access to high-quality ACT through a donor-funded subsidy for ACT and supporting interventions. Results from the AMFm independent evaluation (IE) suggest that the private sector can be an efficient partner to work with and that significant achievements in access to effective malaria treatment can be made in a short period of time [8,9,15-20]. However, a central criticism of the AMFm mechanism was that a substantial share of subsidized, private sector ACT was likely being taken by patients without malaria, since many private outlets do not offer diagnostic testing [21]. The reach of the private sector with respect to ACT treatment—combined with its limitations with respect to targeting of ACT to malaria-positive patients—has fuelled an interest in scaling up diagnostics through private sector channels.

Several pilot programmes have experimented with introducing rapid diagnostic tests (RDTs) for malaria in private sector clinics and drug shops [12,22-24]. RDTs for malaria have attracted interest in recent years because of their high sensitivity, specificity and simplicity for use in resource-poor settings. An RDT requires the tester to collect one drop of blood through a finger prick and place it onto a small plastic device called a cassette, which displays the result of the test within 15–20 minutes. The correct and safe administration of RDTs by non-medical personnel is well documented in the literature [24-29]. RDTs could shrink the gap in diagnostic testing rates by extending parasitological confirmation of malaria to communities without access to microscopy or to areas where barriers to microscopy exist.

Increasing availability of RDTs in the private sector has great potential for improving diagnosis and treatment but introduces a number of challenges on both the supply and demand side. Operational challenges from the supply side include ensuring that private providers transport, store, administer and dispose of the tests and accompanying materials (lancets, etc.) properly [30]. The private sector in most malaria endemic countries is highly variable in quality—from licensed pharmacies and professionally-run clinics to informal drug shops—and notoriously challenging to monitor and regulate. Several studies have demonstrated that private sector providers perform very well in the domains of RDT transport, storage and administration [24,31,32] but have found variation in adherence to the test results with respect to medication dispensing (Obinna Onwujekwe, personal communication;[33-35]). Introducing RDTs into the private sector also raises questions about provider incentives to offer the tests. Cohen and Dickens show in a theoretical model that there can be strong profit incentives to private providers offering RDTs and Hutchinson *et al.* [36,37] show, in a qualitative analysis, that private providers may want to offer the tests because of the reputational benefits and because it allows them to offer a higher quality of care.

Private sector RDT scale up also introduces a number of challenges on the demand side. Patient demand for and valuation of testing—less critical for public sector scale up--become central to private sector introduction. In particular, people have to see the need for testing, the value in it and be willing to pay for it. Generating private demand for public health products can be complex, and techniques for bolstering demand include commercial and social marketing, branding, packaging and behaviour change communication. Encouraging testing for malaria requires overcoming a longstanding belief that all fevers are likely to be caused by malaria, fuelled partly by previous malaria guidelines emphasizing the treatment of all febrile children with anti-malarials [38,39]. More generally, patients, caregivers and health care providers often feel capable of diagnosing malaria based on symptoms, which can diminish the demand for testing.

Existing research on patient and provider perceptions of RDTs available to date has identified a range of sociocultural barriers to RDT uptake, including lack of confidence in test results or the person administering the test, fear of high cost of testing, fear of undisclosed HIV testing, risk aversion to not treating a potential malaria illness coupled with a belief that ACT does not have negative side effects, confidence in oneself to know the signs and symptoms of malaria and properly treat it without medical consultation, and rejection of blood testing as a tool for diagnosis of disease [23,40,41]. Although patients and caregivers often express a high level of enthusiasm for RDTs, trust in the test may be limited by the expectation that the diagnosis will align with the patient’s own self-diagnosis or a belief that the test will result in diagnosis of any illness, beyond just malaria [12,42,43]. This study builds on this literature by focusing on the determinants of and barriers to demand for RDTs in private sector drug shops. It goes beyond assessing feasibility and acceptability of RDTs in drug shops to explore whether the foundational elements of willingness to pay are present in communities in eastern Uganda.

In Uganda, malaria is highly endemic and a leading cause of morbidity and mortality, especially amongst children [44]. Due to the lack of availability and convenience of diagnostic facilities in Uganda, presumptive treatment of malaria is very common [9]. The private sector, including private clinics, pharmacies and small drug shops, serves the majority of people seeking treatment for febrile illnesses in Uganda [9,45-47]. However a recent survey found that only 21% of private, for-profit outlets had malaria diagnostic tests available [8]. In order for Uganda’s National Malaria Control Programme to meet their national target of 90% of all suspected malaria cases to receive confirmatory diagnosis by 2015, use of diagnostic technology for malaria in the private sector will need to be increased.

This qualitative study was conducted as part of a larger project investigating the feasibility and impact of RDT distribution via private sector drug shops in Uganda [24]. As part of the implementation strategy, a behaviour change communication (BCC) campaign was planned to encourage and sustain the demand for RDTs in drug shops in Uganda. While incentive alignment and behaviour change on the supply side are crucial to RDT introduction as well [36], this campaign was focused on demand creation from the patient/community and thus the focus groups probed most specifically into foundational elements of demand. These results describe the findings of a qualitative assessment that was conducted in order to inform and design the BCC strategy.

## Methods

### Study design and questionnaire

The primary objective of this study was to explore the following research question: What are the barriers and incentives to purchasing malaria diagnosis among patients and what recommendations can be made for boosting demand for RDTs introduced into the private sector, particularly in drug shops? Focus group discussions were conducted with community members to explore both community practices and perceptions around malaria diagnosis. The field guide questionnaire included both very open-ended questions such as “What do people in this community think about blood testing for malaria?” and “How do people in your community know when they have malaria?”, and more targeted questions such as “Often people take malaria medicine without being tested. Why?” More targeted questions were derived from an expected utility framework for malaria diagnosis (discussed below) and, combined with open-ended questions, were designed to also uncover additional barriers to demand for RDTs. Broad themes explored included: why people do or do not seek diagnosis for malaria, how people react to test results and why and what can be done to increase the use of diagnostic testing and, in particular, RDTs.

### Sites, sampling and timeline

**Figure 1:**
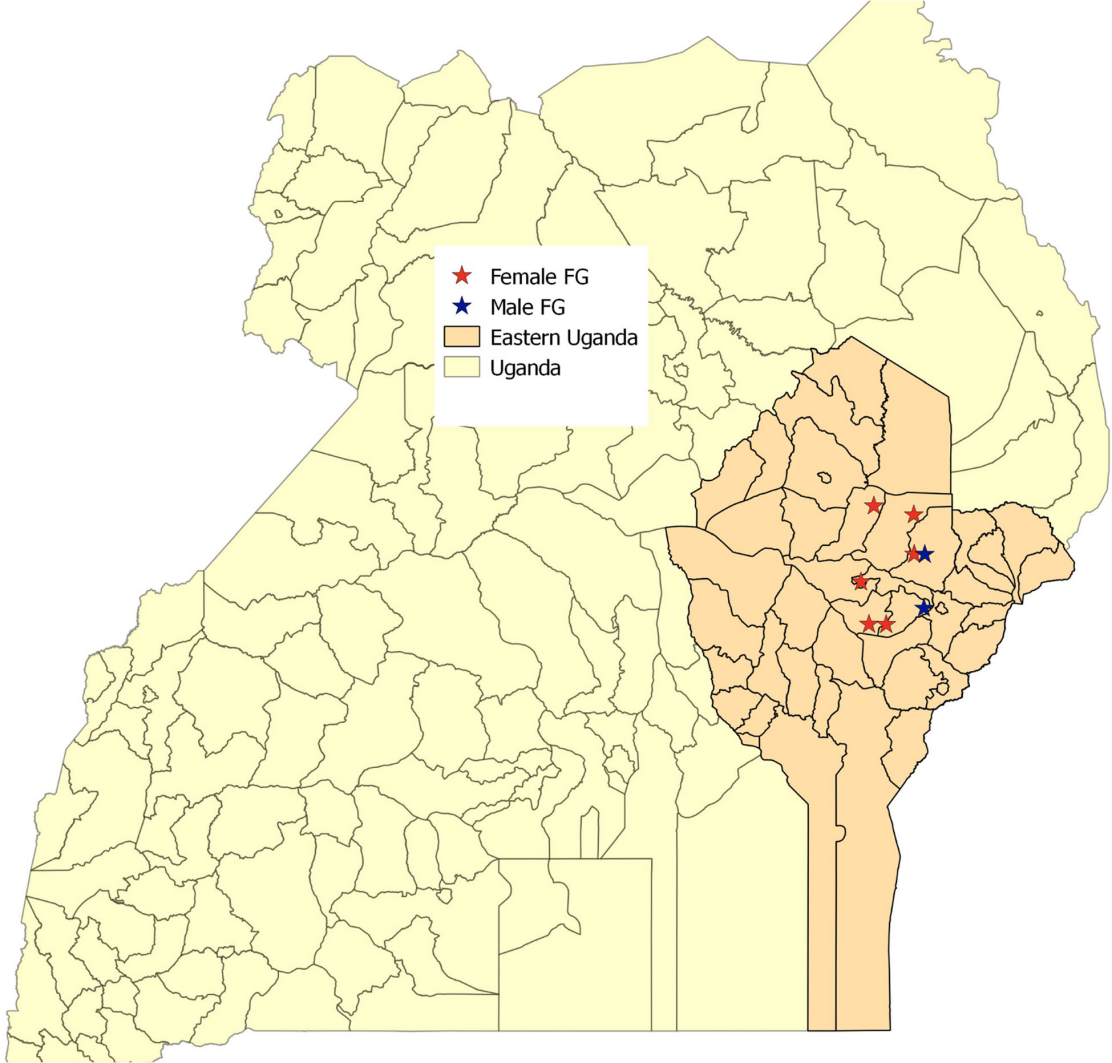
**Spatial location of focus groups.**

A total of eight focus group discussions (FGDs) were conducted in Uganda’s Eastern Province between 28 November and 28 December, 2011. Six districts of eastern Uganda were selected as sites for the study: Budaka, Bukedea, Kibuku, Kumi, Ngora, and Pallisa. Figure 1 shows the spatial location of the eight focus groups.

Six focus groups were conducted with female participants and two with male participants after informed consent was secured. For each focus group, 10–12 participants were recruited with the help of local community leaders, with a particular focus on adults with children under the age of five. For the female focus groups, participants were rural women from age 18 to 50, identified as either pregnant or caregivers of children. Male participants were between age 18 and 56 and either fathers or caretakers of children.

Table 1 summarizes the gender and age distribution of focus group participants. A total of 62 women and 22 men participated in the focus group. Given the explicit focus on participants with children, the majority of participants were age 30 and older; the average number of children in their household was 4.4.

**Table 1:** **Sample composition**

	**Females 15-29**	**Females 30-59**	**Males 15-29**	**Males 30-59**	**Average number of children**
Budaka District FG 1	2	10	0	0	6.2
Budaka District FG 2	0	0	5	7	2.8
Bukedea District FG 1	7	3	0	0	3.7
Bukedea District FG 2	0	0	4	6	4.6
Kibuku District FG 1	2	8	0	0	5.2
Kumi District FG 1	4	6	0	0	3.8
Ngora District FG 1	3	7	0	0	4.4
Pallisa FG 1	4	6	0	0	4.8
**Total**	**22**	**40**	**9**	**13**	**4.4**

### Training, data collection and translation

Field teams were provided with a three-day training on qualitative methodologies and ethical issues. Researchers were divided into teams of one supervisor and two research assistants for data collection; each team was responsible for two focus groups. Qualitative interview guides were prepared in English, then translated into Luganda and Ateso and piloted prior to data collection. Focus group discussions were audio recorded; recordings were then transcribed and translated into English for analysis. All fieldwork was conducted by Uganda Health Marketing Group. While exact transcripts of FGDs were requested, transcriptions provided to the researchers included some places where the translation appeared to summarize responses. In effect, the FGD translations include a combination of exact translated transcript and meaning-based translation.

### Data analysis

Data were analysed using open coding to identify recurrent themes, which were then grouped into categories using axial coding to connect related concepts. Five categories emerged and were framed as “potential barriers to malaria diagnosis,” based on the theoretical framework described in the next section. NVivo version 9 (QSR International) was used to manage the data throughout coding and analysis.

### Analytical framework

The theoretical starting point for this study was an expected utility formulation of demand for RDTs as derived in [36], where demand is a function of the expected benefit minus the expected cost to testing, relative to the expected costs and benefits of presumptive treatment with anti-malarials. This framework is broad enough to incorporate a number of crucial elements of the testing decision: 1) the direct and indirect costs such as the cost of the tests and medicines, the time cost of seeking care, and the health and productivity costs of untreated illness; 2) direct and indirect benefits such as health and productivity gains to wellness and psychological benefits to a confirmed diagnosis; and 3) health beliefs and risk perceptions such as the subjective assessment of the likelihood that an illness is malaria, the belief in the accuracy of the test and the belief that the illness, if found not to be malaria, is something serious that needs treatment. Other utility costs include, for example, fears of testing and suspicion that the test detects HIV. While barriers to and enablers of RDT demand stemming from this framework were explored in the FGDs, the FGDs aimed to elicit additional barriers that are not naturally part of this framework or had not appeared in previous literature through very open ended questions. Based on the theoretical framework and the results of the FGDs, we grouped potential barriers to RDT demand into five broad categories:

### Potential Barrier 1 Lack of understanding about malaria testing

The first potential barrier to patient uptake of malaria diagnosis explored is related to knowledge of the disease and understanding of the role of testing and treatment. In order to seek testing for malaria, a patient must possess a certain amount of knowledge about the disease, including knowing that malaria exhibits recognizable symptoms, is detectable by microscopy or RDTs, and is treatable with appropriate medication. If a patient or caregiver lacks understanding in any of these areas, this could reduce the likelihood of seeking diagnosis for malaria.

### Potential barrier 2 lack of perceived need for diagnosis

The second potential barrier explored is related to a patient’s perceived need for diagnosis. In order for a person to seek testing, s/he must understand that symptoms of malaria overlap with symptoms of other diseases, and must believe that a test is necessary to determine whether or not malaria is present. This requires some degree of uncertainty about one’s malaria status (i.e., not complete confidence in one’s ability to self-diagnose). A perceived need for diagnosis also requires a belief that knowing one’s disease status is important and that there is value in the information provided by the test (e.g. that it could save one from having to seek additional care in the future).

### Potential barrier 3 perception that the test/provider lacks credibility

A third potential barrier to patient demand for malaria diagnosis is the perceived credibility of the test and of the provider. If a patient perceives the test to be unreliable or does not trust the provider to deliver the results honestly, then a patient may lose confidence and avoid testing. If patients perceive the test to be accurate and trust the provider, then they may be more likely to seek testing.

### Potential barrier 4 perception that the cost of testing is too high

The fourth potential barrier to patient adoption of malaria diagnosis is related to the perception that testing comes at too high a cost, both monetary cost and time cost. Simply put, a patient will not seek testing if s/he feels s/he cannot afford it or that it is not worth the cost. The out-of-pocket payment at a private facility, the time and expense of travelling to and waiting at a health facility, and health centre visit fees are all potential deterrents for a patient seeking testing. Even though testing has the potential to save the patient money (by helping to avoid the cost of incorrect medications) and time (by preventing repeat visits for treatment), the perceived up-front monetary and time costs of seeking testing may remain a deterrent.

### Potential barrier 5 fear of testing

The last potential barrier explored is fear of testing, including fear of needles, fear of having blood drawn and fear of privacy violations such as unknowingly being tested for HIV.

### Ethics statement

Ethical approval for this study was given by the Harvard School of Public Health (IRB Protocol # P19371-106) and the Uganda National Council for Science and Technology (Protocol # HS805).

## Results

The results of this study shed light on the five potential barriers to patient demand and willingness to pay for malaria diagnosis noted above. For each potential barrier, obstacles that have been overcome and obstacles that remain were differentiated.

### Potential barrier 1 lack of understanding about malaria testing

FGDs indicated that knowledge of malaria, its symptoms and its ability to be treated is common throughout the community. In every focus group, respondents identified malaria as one of the most common illnesses in the community. Respondents noted a wide range of symptoms thought to be associated with malaria, including fever or high body temperature, headache or body pain, lack of appetite, weakness or tiredness, nausea, vomiting or diarrhoea, chills or coldness, and cold-like symptoms including runny nose, coughing or sneezing.

*The most common illness here is malaria.* – 30-yr-old female, Budaka

*A person [who has malaria] will feel a lot of coldness, high fever, body and joint pains, running nose, body weakness and no appetite for food.* – 28-yr-old female, Kumi

*If you see a child with persistent high body temperature and feeling cold, having no appetite and shivering then you will know for sure that child has malaria*. – 25-yr-old male, Bukedea

Participants also showed a general familiarity and comfort with malaria testing as a common part of treatment, and demonstrated good knowledge of where testing is available in their communities. Common places to obtain testing and treatment for malaria were government health facilities and private clinics.

*Last time I had malaria I first felt headache and lost appetite, then felt general body pain and body weakness. When I went for a test it was malaria.* – 50-yr-old female, Pallisa

*Most of us go to the government health centre for blood testing.* – 20-yr-old female, Bukedea

*Those who have money go to clinics to test for malaria*. – 24-yr-old female, Pallisa

Although these results indicate that knowledge about malaria testing and treatment is widespread, one remaining obstacle to ensuring community understanding of malaria testing is a general lack of familiarity with RDTs. The majority of focus group participants had never heard of RDTs, with the exception of four individuals (out of 80 total participants). Those few who had heard of RDTs (typically referring to them as “malaria strips”) knew that they were being used in private clinics to test for malaria and that test results were available in about 10–25 minutes. Overall, participants seemed to have difficulty distinguishing between RDTs and microscopy, most likely because both involve collecting a drop of blood and placing it on a medical device.

*We have never heard of rapid diagnostic tests. We only know the one where they prick and put blood on the slide.* – Budaka

*Is rapid diagnostic test for malaria a person, or what is it?* – 50-yr-old female, Kibuku

### Potential barrier 2 lack of perceived need for diagnosis

The study showed that community members understand that malaria has similar symptoms to a number of other diseases. Specifically, malaria was described as having similar symptoms to HIV/AIDS, measles, and typhoid, as well as tuberculosis, syphilis and cold/flu.

*"Moderator: What other illnesses could it be when it seems to be malaria?*

*Respondent 4: Typhoid is an illness which has all symptoms like those of malaria because you will have continuous headache, vomiting and lack of appetite.*

*R10: Measles also is like malaria in that when a child has measles s/he will have no appetite, will have general body weakness, headache, vomiting and even diarrhoea, and all these are similar to malaria symptoms.*

*R6: Even HIV/AIDS brings up symptoms like those of malaria. A person will feel persistent headache, feeling cold, having high body temperature and lack of appetite.”*

*–* Female FGD, Pallisa

Many respondents described experiences in which they thought they had malaria but it turned out to be something else. The practice of self-diagnosis was a recurrent theme across focus groups, and many people described self-medicating when they exhibited familiar symptoms that they had associated with the disease in the past. In many cases, participants described having bought medication that proved ineffective in relieving their symptoms, and in turn described numerous courses of action they would take, such as visiting a different clinic, requesting a blood test, or visiting a traditional healer. Participants in every focus group said that they had had the experience of thinking they had malaria because the symptoms were familiar and then discovering that they did not have it after being tested.

*I used to have a constant headache and I thought it was malaria but when I tested it was typhoid.* – 20-yr-old female, Pallisa

*I have had a personal experience in this. I had symptoms of malaria and went to test for malaria parasites but the health workers told me that there was nothing. I have also seen some other people who thought they had malaria when it wasn’t*. – 26-yr-old male, Budaka

*I will now go to the doctor so that they will do blood testing to find out what sickness it really is, whether it’s malaria or not since I have previously been taking drugs not knowing what I am suffering from.* – 30-yr-old female, Budaka

*I only begin worrying if the malaria treatment does not heal me, then I think it may not have been malaria…if I was given medicine from the clinic I will change and go to the government health facility for the test.* – 19-yr-old female, Bukedea

*If I see that I am not getting better, I will go for a blood test for malaria before I continue with any treatment.* – 45-yr-old female, Budaka

Community members expressed a general belief that it is important to get tested for malaria before starting treatment. They explained that testing is important in order to know which disease is present and to obtain the correct medication rather than wasting time treating the wrong disease and thereby prolonging illness. Some people even expressed concern regarding the practice of self-medication, which shows again that community members are aware that self-diagnosis can fail.

*It’s good to test blood first before you get treatment because after testing you will know the right treatment to take rather than guessing.* – 33-yr-old female, Bukedea

*One bad thing of not testing for malaria is you will waste time treating what you don’t know and you won’t get well.* – 50-yr-old female, Pallisa

*There is a problem of self-medication where most people say they know their sickness hence no need to test.* – 30-yr-old male, Bukedea

Beliefs about the effects of taking anti-malarials when the disease is not present were mixed. While some participants explained that it is a good idea to take anti-malarials as prevention for malaria, many people mentioned potential dangers of taking malaria medications if the disease is not present, including side effects, disease, overdose and death.

*It is good to take medicine as a preventive measure against malaria.* – 50-yr-old female, Kibuku

*“M: Can you think of some negative or positive effects of taking malaria medicine if you don’t have malaria?*

*R4: For me taking malaria medicine when you don’t have malaria brings nausea and at times vomiting and dizziness. I have a personal experience with this when I tested negative for malaria and health providers gave me medicine to take. I really had a bad experience.*

*R1: When a person keeps on taking drugs when s/he does not have the sickness, it will weaken the immunity in his body such that next time when s/he falls sick, the drugs may not work because s/he has been previously using them even when s/he is normal. Not only that but when you fall sick next time you will be put to another level of treatment.*

*R10: The bad thing of swallowing drugs when you’re not sick is that your body is affected in a way that you become immune to anti-malarials.*

*R5: For me I see that when you swallow drugs [when you do not have malaria], you will put your life at risk. Anything can happen to you as a result of taking those drugs.*

*M: Anything else gentlemen? I have not heard anyone mention any positive reasons to taking malaria medicine when you don’t have malaria.*

*R(ALL): I don’t really think there is anything positive about it because one’s life will be put in danger.”*

- Male FGD, Budaka

Interestingly, most participants also stated that there are scenarios when malaria testing is not necessary. In cases of severe symptoms, such as when a child falls very ill in the middle of the night, participants stated that they would be taken to the hospital for immediate treatment, and that often the provider would skip testing in order to expedite treatment. This would be the appropriate protocol in cases of suspected severe malaria. Participants also described that there is no need for testing when the symptoms are familiar and they are confident that they have malaria. One participant also mentioned that testing is unnecessary during the dry season when mosquitoes are not present.

*People often get signs of malaria whenever they have it, so you find that they don’t bother to test themselves because they always know when they have malaria*. – 34-yr-old male, Budaka

*Normally when you go to the health centre when the child is very ill, they will be put on a drip immediately and test for malaria later.* – 26-yr-old male, Budaka

Despite a general community understanding that malaria symptoms can appear similar to those of other diseases and a belief that knowing one’s disease status is important, people often still self-medicate and wait to see if their treatment is effective before seeking testing. Even though many people have had the experience of misdiagnosing themselves with malaria and only learning their true diagnosis after testing, they often rely on testing only as a secondary strategy.

### Potential barrier 3 perception that the test/provider lacks credibility

Respondents seemed to reflect a confidence in diagnosis-based treatment at public facilities, which were generally preferred over private clinics. Government health facilities were preferred for what was perceived to be honest and well-trained staff, high quality drugs and services, and referral capabilities. Respondents mentioned that government facilities give the correct treatment, do not provide expired drugs, and provide more effective drugs, as compared with private clinics. Participants also mentioned that the government health facilities are able to refer patients to bigger hospitals when the condition is serious. While respondents generally seemed to trust government health facilities, results of the study showed some degree of distrust in private providers. In a few instances, participants reported that private clinics were dishonest and would sell expired medications or lie about test results in order to sell more medications and increase profit.

*“M: Which places do you trust most?*

*R9: I trust government health facilities because health provides there are always honest and trained but in clinics since it’s business they can lie that you have malaria because they want money.*

*R3: I trust government health facilities because if they have drugs they can give for free.*

*R2: I trust government health facilities because if they have trained staff they also have better equipment and if you condition is worse they can refer you to a hospital.*

*M: Anything else?*

*R10: We always use both clinics and government health facilities.”*

- Male FGD, Bukedea

Participants described malaria diagnosis as typically consisting of the provider taking an oral history, performing a physical examination, taking vitals, and ordering a blood test for malaria. Most participants agreed that if a provider does not test for malaria during a visit, it is due to the fact that there is no indication of malaria. In most cases, providers were reported to have explained test results to patients. However, a few respondents described receiving a blood test and being given medications without ever being told whether or not they in fact have malaria.

*In the consultation room the health provider will first take your history – how old are you, what is the problem, when did it begin – and sometimes he checks you using a thermometer then later sends you to the laboratory for a blood test.* – 40-yr-old female, Pallisa

*If you get a good doctor, he will tell you what your illness is but others will just treat you and you go back home without knowing the disease.* – 26-yr-old female, Kumi

A few participants also reflected a possible lack of confidence in test results. Many respondents discussed having taken malaria medications even after receiving a negative blood test result, either because the provider still prescribed it or because they still felt sick and bought medication anyway. In this scenario, participants cited various perceived benefits of taking medication, including prevention of malaria and “just in case” malaria is “hiding” in the body. The desire to take medication in case the test failed to detect malaria suggests a belief that the test is not always effective at detecting malaria. Perceived credibility of the provider and test was mixed. While participants generally expressed trust in public facilities, there was some degree of distrust in private facilities. Some patients also questioned the reliability of the test, citing experiences when they took anti-malarials even after a negative test result, reasoning that the malaria could be “hiding.”

*I was tested and found that I do not have malaria but I was again given drugs reasoning that the malaria might be hiding.* – 30-yr-old female, Kumi

*When you are not feeling well and you test negative you will still have a feeling that malaria might be hiding so you continue taking malaria drugs.* – 37-yr-old male, Bukedea

### Potential barrier 4 perception that the cost of testing is too high

Perceptions of time and monetary costs were reflected in patients’ preferences for seeking care at government health facilities *versus* private clinics. Results indicated that the monetary cost of testing, including fees for visits, supplies, medical record books, and the test itself, remains an important barrier for patients seeking testing. Respondents described private clinics as being the most expensive option.

*If you don’t have money no clinic can ever give you a free test.* – 26-yr-old female, Pallisa

*In clinics when you go they ask you what the problem is and if it is malaria related they ask if you have money for a blood test.* – 19-yr-old female, Bukedea

Many participants preferred seeking care at government health facilities due to the fact that they provide free services. However, in some cases, government health facilities were also faulted for charging fees even though user fees were abolished in the Ugandan public health system in 2001 [48]. In one district, numerous respondents mentioned that at government health facilities they were asked to buy syringes and gloves in order to receive testing for malaria.

*People prefer going to a government health facility because they get free treatment and they are given right dose of treatment.* – 21-yr-old female, Bukedea

*Sometimes you go to the health centre and find that they need money for the record book and for testing blood and yet you do not have the money.* – 38-yr-old female, Ngora

*When you go to the government facility and you are sent for a blood test the laboratory assistant will ask you for money for buying gloves and syringe for testing your blood and if you do not have it you are told to go back home then come back.* – 43-yr-old male, Bukedea

Time costs were equally as important as monetary costs, and were reflected in a different set of preferences regarding providers. Respondents described multiple time-related barriers to accessing care at government health facilities, including distance and long lines/wait times. Government facilities were described as being farther away and more difficult to reach on foot as compared to private clinics. Many patients described difficulties in reaching these facilities due to transportation costs and the burden of carrying small children along the way. Additionally, long lines and wait times were common and sometimes prohibited people from accessing care. Many respondents mentioned that the health centres often experience drug stock-outs and refer patients to private clinics to purchase medicines, an additional delay for patients accessing care.

*It takes six miles to reach the health facility and when you reach the hospital the line is long so you spend the whole day in the hospital without getting treatment.* – 50-yr-old female, Kibuku

*These days I just go to the clinic to test and get treatment because when you go to the health centre they only prescribe treatment for you and they tell you that they don’t have drugs and then they send you to the clinic to buy the drugs.* – 26-yr-old female, Pallisa

In contrast, private clinics offered faster service, longer hours, and close proximity to the community, all factors that help patients to save time. Participants described that while government health facilities can have long lines and long wait times, at private clinics patients receive care without delay. They mentioned that sometimes government facilities open late or close early, while private clinics are generally open longer hours on a more consistent basis.

*I trust private clinics. They are always conscious about time – they don’t keep you waiting for long. They also they know how to monitor and care for their patients.* – 26-yr-old male, Budaka

*If you are badly off sometimes you will go to the health unit and find that there are very many people, so in order not to wait you will go to the clinic which also does testing and there aren’t very many people so you will be attended to immediately.* – 50-yr-old female, Ngora

Patients described prioritizing treatment over testing when faced with limited resources. In many cases people preferred to spend what money they had on medications rather than testing, for fear that purchasing a test would prohibit them from being able to afford the necessary treatment. Many people also said that they were unable to make the journey to the health centre and could not afford transportation to get there, so it was easier to buy medications at a local clinic or drug shop. There was no mention of the possibility that testing could save money by potentially avoiding the cost of inappropriate medications.

*Lack of money is what makes people get treatment before testing…a person will debate that they only have a little money and if they waste it on testing blood, then they will not have enough money for drugs…people prefer buying drugs.* – 37-yr-old male, Bukedea

*The health centres which test are very far and sometimes there is no money for testing. So, if you are badly off you will first seek treatment before testing from the nearest clinic.* – 31-yr-old female, Kumi

*For example I have two young children, one age one year and another three years. It is difficult to carry both to go to the health centre so I end up treating them without testing.* – 36-yr-old female, Budaka

Cost, both time and monetary, remains a complicated and important barrier to malaria testing, as all participants cited a variety of testing fees, transportation costs and time costs that influence their decision to seek malaria testing. Generally financial costs were lower at public facilities, which were more likely than private clinics to provide free services. However, private clinics were more easily accessible than public facilities, therefore presenting a lower time cost. Faced with limited resources, patients were likely to pay for medications before testing.

### Potential barrier 5 fear of testing

While no fear of needles or blood drawing emerged from the focus groups, some participants expressed fear that a provider would use the blood test to perform HIV testing without consent. In addition to exposing the fear of knowing one’s HIV status, this example also reflects a degree of distrust in providers and highlights a remaining barrier, ensuring patient confidentiality in all testing venues.

*Some people think that when they go get tested for malaria in the health centre, they will also be tested for other diseases like AIDS. So they prefer taking drugs rather than getting tested and knowing their HIV status.* – 50-yr-old female, Ngora

*Some people fear to know the results of the blood test as if it is an HIV test.* – 50-yr-old female, Kibuku

## Discussion

The scale up of diagnostic testing is now a central component of malaria policy and is a cornerstone of effective and efficient case management. For some countries and contexts, the private sector will likely not play a major role in this scale up, as treatment-seeking in this sector is minimal and/or the challenges to private sector engagement (regulation, monitoring, etc.) are currently too substantial. In other countries, however, where there is heavy reliance on the private sector for malaria treatment, strategic engagement of private sector providers will be essential to the ability to attain universal access to diagnostic testing. Overall, the effectiveness and cost-effectiveness of private sector RDT strategies will depend on a country’s endemicity and treatment-seeking profile [49]. A recent Cochrane Review of the impact of RDTs on fever case management came to the conclusion that, while RDTs have great potential to reduce unnecessary usage of anti-malarials, they may not lead to significant improvements in morbidity and mortality [50]. The economic and health impact of RDTs will clearly depend crucially on the disease profile and the treatment-seeking behaviour of local populations.

Several pilot programmes have been implemented recently or are underway exploring the feasibility and impact of private sector provision of RDTs in Africa. Short of making testing in the private sector a legal requirement (which could be excessively difficult and expensive to enforce in a number of countries/contexts), successful scale-up of RDTs in the private sector will require that patients view RDTs as a valuable commodity, with credible and useful information that can be used to guide treatment and for which they are willing to pay, despite very limited resources. This study was designed to explore potential barriers to uptake of malaria diagnosis in eastern Uganda with the objective of understanding how to design successful behaviour change campaigns and supporting interventions for programmes and policies scaling up private sector RDT distribution.

Participants mentioned several reasons why they felt diagnosis was important. One reason was that symptoms of non-malarial illnesses can appear like malaria. Participants noted times when they thought they had malaria and discovered they were wrong. The belief that one can be wrong in his or her assessment that an illness is malaria is a critical precondition for valuing the RDT. A common concern in scaling up malaria diagnosis is that people will not value or accept the test because they are very confident in their ability to self-diagnose based on symptoms (especially fever). While participants did seem to place quite a bit of confidence (perhaps overconfidence) in their ability to know when an illness is malaria, overall the results from our focus group discussions suggest that people are aware that they can be mistaken and do feel uncertainty in their assessments about malaria status. Emphasizing that not all fevers are malaria thus may be necessary but not sufficient foundation for a BCC strategy. Some communities may already appreciate the overlap between malaria and other illnesses and may be choosing to forego diagnosis for other reasons.

Another common concern regarding potential demand for malaria testing is that the perceived costs of taking malaria medicine when it is not needed are low. The more concerned patients or caregivers are about the costs of taking unnecessary medication, the higher the valuation of an RDT should be. A number of participants mentioned potential dangers of taking anti-malarials when they are not needed, including side effects. While side effects did seem to be a concern, participants did not mention other potential costs of taking unnecessary medication. This is somewhat surprising, considering how resource constrained these households are. Participants also did not mention concerns about unnecessary medication leading to delays in treatment for the true cause of illness. While it is possible that these potential benefits of RDTs have simply not been considered in these communities, it suggests that these themes may not resonate in certain communities and may be ineffective as cornerstones to a BCC strategy emphasizing malaria testing.

Overall, participants were very familiar with malaria diagnostic testing and where testing could be obtained. Despite a familiarity with testing and knowledge of its importance, the prevailing community practice is typically to seek testing only when presumptive treatment proves ineffective. This has been found in other recent qualitative research on RDTs in Uganda [23]. The use of testing as a secondary strategy suggests that neither the perceived benefit of diagnosis, nor the perceived cost of incorrect treatment, is significant enough to lead a patient or caregiver to seek testing *first*, although apparently the benefits of diagnosis appear more salient to patients after presumptive treatment has failed. This suggests that the monetary and time costs associated with testing are significant barriers to demand for malaria diagnosis. Many participants mentioned the difficulty and inconvenience of testing. Taken together, this suggests that BCC campaigns may want to emphasize the convenience and ensure the affordability of RDTs in the private sector, their proximity to households, the speed with which results can be acquired, etc.

The focus groups showed that when faced with limited financial resources, patients generally prefer to spend their money on treatment rather than testing. They expressed concern that they may be unable to afford treatment if they purchase a test first. Even in public facilities, where testing and treatment are supposed to be free in Uganda, fees are often charged for testing equipment (e.g., gloves and syringes) and medications can be stocked out, requiring the patient to purchase medication in the private sector. Transportation costs, wait times and convenience were also drivers of individual decision-making. In the case of a young child falling ill in the middle of the night, a parent might accept higher fees at a drug shop because it is more convenient given the urgency of the situation.

An important finding to keep in mind is that despite widespread knowledge of malaria testing, familiarity with RDTs was limited. This suggests, and may be due to the fact, that many people have not encountered RDTs. In a household survey conducted for a different aspect of this project with a representative sample of the local population (see [24]) only 25% of respondents had heard of RDTs. Public health facilities in the area were also surveyed as part of this project and, at the time of the focus groups, roughly 50% of facilities had RDTs in stock. Thus, while RDTs were a somewhat new technology at the time, they had already been rolled out to a significant share of public facilities though were very rarely available in drug shops. Very few participants had actually heard of RDTs, so the majority of opinions about malaria testing were based on experiences with what was perceived as microscopy – in practice distinguishing RDTs from microscopy may not be easy for patients due to the common finger-pricking procedure. While the focus groups showed that patients are sometimes sceptical of test results, and occasionally continue taking anti-malarials after a negative test, it is important to keep in mind that these opinions were generally based on experiences with microscopy.

Despite the remaining barriers, a general lack of familiarity with RDTs may actually present an opportunity to tip the scale towards adoption of RDTs. Interventions promoting RDT sensitization and education, combined with increasing the availability and affordability of RDTs in private sector drug shops, could help to promote RDTs as a reliable, inexpensive and convenient source of diagnosis. A patient who prefers not to seek testing due to the long travel and wait times in the public sector may be pleased to learn that testing by RDTs eliminates both of those barriers. However, sensitivity to cost must be kept in mind. Both because people may have limited financial means and because ACT is heavily subsidized in many places, it may be appropriate and necessary to heavily subsidize RDT sales in the private sector [36].

The results in this study may not be generalizable across all contexts. First, the level of malaria prevalence is quite high in this region (nearly 50%) [34]. A high level of malaria prevalence to some extent decreases the value of the test, since the likelihood that the test can allow the patient to avoid unnecessary medications is lower. If people in this community are used to testing positive, they may see less reason to continue being tested in the future. On the other hand, this study and others [23,51] have found that patients place a high value on knowing the source of illness, particularly if it confirms their expectations. A positive RDT result would enable a patient or caregiver to be sure that she was treating the correct illness and in that sense may increase valuation of the test. If positive results build confidence in and valuation for testing, RDTs may actually be more successfully introduced to high endemicity settings.

Further, the FGDs took place during the AMFm pilot programme in which ACT was heavily subsidized in the private sector and drug shops. This programme substantially reduced the price of ACT, the recommended first-line treatment for malaria, and made it much more widely available in this area [8,15]. The value of a diagnosis could also be reduced when ACT is more affordable [36]. Finally, patient demand for RDTs could depend substantially on the major causes of non-malarial illnesses in an area. If people tend to presumptively treat for malaria and then test only if treatment fails, they will most often never need to test if the illness was either malaria (as it is in high prevalence settings) or was a simple self-limiting virus. These types of illnesses that will simply resolve on their own over time tend to be the major cause of non-malarial fever [52].

## Conclusions

Results from our FGDs suggest that, at least in contexts such as eastern Uganda, private sector RDTs will have to be made affordable and convenient in order for a substantial share of patients to pursue testing as a first course of action for suspected malaria. This may mean subsidizing the tests and distributing them through retail establishments that reach remote areas, so the tests can be as accessible and convenient as possible to rural communities. Enhancing the credibility of providers, for example, through accreditation programmes, could help communities trust test results from these establishments. Private sector RDT programmes may need supporting interventions, such as subsidies and behaviour change campaigns in order to reach substantial scale. It is possible that these interventions need only take place early in the programmes as demand for RDTs grows. Overall, private sector markets and foundational elements of demand for testing are likely to vary widely across malaria-endemic settings, and more research is needed to understand how to boost testing throughout malaria vulnerable populations.
